# OXPHOS Organization and Activity in Mitochondria of Plants with Different Life Strategies

**DOI:** 10.3390/ijms242015229

**Published:** 2023-10-16

**Authors:** Irina V. Ukolova, Gennadii B. Borovskii

**Affiliations:** Laboratory of Physiological Genetics, Siberian Institute of Plant Physiology and Biochemistry, Siberian Branch of the Russian Academy of Sciences, 664033 Irkutsk, Russia; borovskii@sifibr.irk.ru

**Keywords:** BN-PAGE, OXPHOS, supercomplexes, plant mitochondria, maize, pea, winter wheat

## Abstract

The study of the supramolecular organization of the mitochondrial oxidative phosphorylation system (OXPHOS) in various eukaryotes has led to the accumulation of a considerable amount of data on the composition, stoichiometry, and architecture of its constituent superstructures. However, the link between the features of system arrangement and the biological characteristics of the studied organisms has been poorly explored. Here, we report a comparative investigation into supramolecular and functional OXPHOS organization in the mitochondria of etiolated shoots of winter wheat (*Triticum aestivum* L.), maize (*Zea mays* L.), and pea (*Pisum sativum* L.). Investigations based on BN-PAGE, in-gel activity assays, and densitometric analysis revealed both similarities and specific OXPHOS features apparently related to the life strategies of each species. Frost-resistant winter wheat was distinguished by highly stable basic I_1_III_2_IVa/b respirasomes and V_2_ dimers, highly active complex I, and labile complex IV, which were probably essential for effective OXPHOS adaptation during hypothermia. Maize, a C4 plant, had the highly stable dimers IV_2_ and V_2_, less active complex I, and active alternative NAD(P)H dehydrogenases. The latter fact could contribute to successful chloroplast–mitochondrial cooperation, which is essential for highly efficient photosynthesis in this species. The pea OXPHOS contained detergent-resistant high-molecular respirasomes I_1–2_III_2_IV_n_, highly active complexes IV and V, and stable succinate dehydrogenase, suggesting an active energy metabolism in organelles of this plant. The results and conclusions are in good agreement with the literature data on the respiratory activity of mitochondria from these species and are summarized in a proposed scheme of organization of OXPHOS fragments.

## 1. Introduction

The provision of aerobic eukaryotic cells with energy and Krebs cycle intermediates, maintenance of mitochondrial biogenesis processes, and regulation of signaling pathways are carried out through the organized operation of the oxidative phosphorylation system [1]. This multi-enzyme and multifunctional machine is localized in the inner mitochondrial membrane and includes complexes I (NADH-ubiquinone oxidoreductase; EC 7.1.1.2), II (succinate-ubiquinone oxidoreductase; EC 1.3.5.1), III (ubiquinol-cytochrome *c* oxidoreductase; EC 7.1.1.8), and IV (cytochrome *c* oxidase; 7.1.1.9) of the respiratory chain; phosphorylating complex V (F_o_F_1_-ATP synthase; EC 7.1.2.2); mobile electron carriers, ubiquinone and cytochrome *c*; and ATP synthase coupled carriers of adenine nucleotides and inorganic phosphate [2,3]. The components of the OXPHOS constitute the bulk of the inner membrane proteins and, according to various data, occupy from 46% to 70% of its hydrophobic volume, which is significantly higher than the protein content in other cell membranes (reviewed in [4]). Higher-order spatial organization is necessary for enzymes at such high concentrations to work efficiently and to prevent a number of negative consequences, such as nonspecific protein aggregation, bilayer deformation, and anomalous diffusion. 

Multiple proteomic and electron microscopy studies have confirmed the supramolecular organization of OXPHOS components. It has been shown that respiratory supercomplexes with different stoichiometries and dimer ribbons of ATP synthases are present in the inner mitochondrial membrane of various organisms (discussed in [5]). There are four main superstructures found in these organisms that have been well characterized using cryo-electron microscopy, namely respiratory supercomplexes I_1_III_2_, III_2_IV_1–2_, and I_1_III_2_IV_1–4_ and dimeric ATP synthase [6,7,8,9,10,11,12,13,14,15,16]. The supercomplex I_1_III_2_IV_1–4_ is called a true respirasome [17], and also a N-respirasome [18], because it contains all the necessary molecular elements (complexes I, III_2_, IV, CoQ, and cyt *c*) for autonomous respiration on NADH. The available structural data demonstrate that respiratory chain supercomplexes have a conserved core of complexes I and III_2_ [11]. Differences concern complex IV arrangement [11] and the angle between the complex I membrane arm and complex III_2_ (from the matrix view) [15]. For example, plant supercomplex I_1_III_2_ has been shown to be more open in comparison with the tighter mammalian counterpart [15]. In addition to those described above, other associations of electron transport chain enzymes have been detected. There are also data for complex II incorporation into respiratory supercomplexes (reviewed in [19]). Apart from this, a phosphorylating supercomplex called ATP-synthasome consisting of ATP synthase, phosphate and adenine nucleotide carriers was revealed [20,21]. Specific structural and functional advantages have been attributed to the supramolecular organization of the OXPHOS complexes, namely (i) efficient substrate channeling, (ii) prevention of ROS generation, (iii) structural stabilization and interdependent biogenesis of individual respiratory complexes in respiratory supercomplexes [22,23].

Nevertheless, the physical and functional organization of the OXPHOS in vivo still remains unclear. Biochemical studies using BN (Blue Native) electrophoresis techniques showed that detergent solubilization of mitochondria led to the isolation of both supercomplexes and free OXPHOS enzymes, the ratio of which changed depending on cell type and the organism’s physiological state. These data contributed to the emergence of a “plasticity” model [17], according to which, free respiratory complexes and a varied set of supercomplexes coexist in vivo and their relative number varies between cell types and changes in response to physiological stimuli [22]. A number of studies have suggested that respiratory supercomplexes can associate with each other to form even more higher-ordered structures in vivo, such as respiratory strings and patches [8,24,25,26,27]. At the same time, facts are gradually accumulating that indicate the possibility of physical interactions not only between respiratory complexes but also between ATP synthase and respiratory enzymes (described in [19,28]). Based on these data and taking into account the structural and functional division of the inner membrane into subcompartments, a subcompartmented oxphosomic organization of the OXPHOS was recently proposed [19]. This implies that part of the respiratory supercomplex population physically interacts with oligomeric ATP synthase to form oxphosomes, which are located predominantly in the mitochondrial cristal subcompartment as highly organized sites, which in fact represent “mini-factories” for ATP production. The rest of the respiratory supercomplexes and individual complexes can remain in a free form, and the degree of higher-ordered OXPHOS superassembly (i.e., the abundance of highly organized OXPHOS patches and strings) probably depends on the organism, cell type, physiological status, and energy requirements. It is assumed that part of the supercomplex population solubilized with detergent is the breakdown products and basic “building blocks” of oxphosomic strings and patches.

Plant mitochondria have a branched electron transport chain owing to the presence of numerous alternative oxidoreductases [29] and differ in the lower abundance of respirasomes compared to mammalian and fungal counterparts [30,31]. Many studies of OXPHOS organization in plant mitochondria have observed only the most stable supercomplex I_1_III_2_ [32,33,34,35]. Nevertheless, N-respirasomes I_1_III_2_IV_n_ and supercomplexes III_2_IV_1–2_ were found in significant amounts in potato [36], spinach [30], and pea [19]. It was assumed that higher yield of intact respirasomes was associated with higher integrity of solubilized organelles. Recently, using BN-based electrophoresis techniques, we have managed to detect both the main supercomplexes routinely observed in various organisms and new OXPHOS structures, which have not been detected in other plant species or even other organisms, in particular, megacomplex (II_x_III_y_IV_z_)_n_ and supercomplex IV_1_Va_2_. The latter included a dimer of a recently detected form Va of ATP synthase [19]. 

The present research aimed to further investigate the features of supramolecular and functional OXPHOS organization in the mitochondria of various plant species and to analyze the possible link between these features and biological characteristics of the species. For this purpose, the composition and activity of the OXPHOS components solubilized from pea, winter wheat, and maize shoot mitochondria were studied using 1D BN-PAGE in combination with an in-gel activity assay and a densitometric analysis. The selected species belong to different families (pea—Fabaceae; winter wheat and maize—Poaceae) and classes (Dicotyledonous and Monocotyledonous) and have distinct life strategies. The results showed the highly conserved architecture of the OXPHOS in these plants and also revealed the specific OXPHOS features apparently related to the life program of each species. Notably, the OXPHOS in pea mitochondria was characterized by detergent-resistant high-molecular respirasomes that could indicate its higher-ordered or/and more stable native organization in comparison to that in cereals. In addition, stable complex II and highly active complexes IV and V suggested an active energy metabolism in this plant (discussed in Section 3.4). One of the most prominent features of the winter wheat OXPHOS was the highly active complex I. We assumed that such a high activity of this enzyme was essential for cold adaptation and survival of this cereal (Section 3.2). It should be noted that the activity of winter wheat complex I in BN gel was in good agreement with its respiratory activity in organello, which was assessed by complex I substrate oxidation (Section 2.1.1). In maize, the lower complex I activity was obviously compensated for by the higher activity of alternative NAD(P)H-dehydrogenases, which could be required for maintenance of intensive photosynthesis in this plant (Section 3.3). Furthermore, cereals differed in more stable respirasomes I_1_III_2_IVa/b and dimers V_2_, and maize also had very stable dimers of complex IV. The significance of the revealed features of the OXPHOSs for the life program implementation of each species is discussed here taking into account the available literature data and in the light of the oxphosomic organization suggested earlier. 

## 2. Results

As noted above, the species of interest belong to distinct families and have radically different life strategies. Thus, winter wheat is a frost-resistant cereal, which can withstand significant temperature fluctuations and survive winter in Eastern Siberia. The LT_50_ (lethal temperature for 50% of the samples) for hardened winter wheat seedlings is −14.6 °C [37], and plants at the crown stage tolerate a decrease in temperature at the tillering zone to −16…−18 °C. In contrast, maize, a C4-type heat-loving plant with highly efficient photosynthesis, differs from winter wheat in a much narrower temperature adaptation range, and is sensitive to frost and tolerant to high temperatures [38]. In terms of temperature tolerance, cold-resistant pea is intermediate between these cereals. In addition, this plant is a nitrogen fixer; its roots enter into an elaborate symbiosis with rhizobial bacteria that enables the production of protein-rich seeds. These features require extra energy and, consequently, active involvement of mitochondria that, in turn, can be reflected not only in the organization of the energy system of root cells [33], but also of the whole plant.

**Figure 1:**
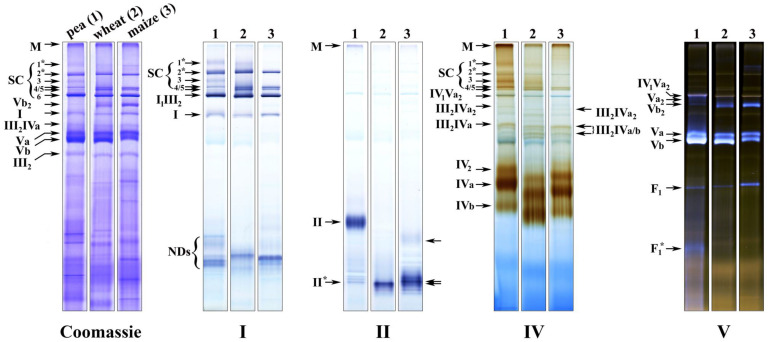
Comparison of complement and activity of the OXPHOS components from pea, winter wheat, and maize shoot mitochondria. Left panel—Coomassie-stained gel strips with digitonin-solubilized mitochondrial complexomes from pea, winter wheat, and maize shoots. I, II, IV, V—in-gel activity assay for complexes I, II, IV, V, respectively. The components of the OXPHOS are indicated on the left and on the right, where necessary. M is megacomplex (II_x_III_y_IV_z_)_n_. SC_1–6_ are supercomplexes with the putative composition: SC_1_—I_2_III_2_IV_n_, SC_2_^*^—I_2_III_2_ + SC^IV^, SC_3_—I_1_III_2_IV_n_, SC_4_—I_1_III_2_IVa, SC_5_—I_1_III_2_IVb, SC_6_—major supercomplex I_1_III_2_ + minor IV_1_Va_2_ [19]. I, II, III_2_, IVa, IVb, IV_2_, Va, Vb, Va_2_, Vb_2_—the corresponding complexes, their forms, and dimers. II^*^—breakdown products of complex II. F_1_ and F_1_^*^—the F_1_ component of complex V and its breakdown product, respectively. III_2_IVa/b_1–2_—pea supercomplexes III_2_IVa_1–2_ are marked on the left, and cereal supercomplexes III_2_IVa/b and III_2_IVa_2_ are marked on the right, of which III_2_IVa_2_ is detected in trace amounts under the conditions applied. NDs—alternative NAD(P)H-dehydrogenases. The arrows on the right side of the gel II indicate the major forms of the wheat and maize enzyme and the minor form of the maize one (Table S1). SC_1_^*^—as distinct from winter wheat and pea, maize SC_1_ with the calculated mass of 2760 kDa (migrated just below pea SC_1_) showed only ATPase activity and was most likely an oligomeric form of ATP synthase (Figure S1). SC_2_^*^—instead of the earlier assumed respirasome I_2_III_4_IV_n_ [19], two associations are supposed to comigrate in this band: I_2_III_2_ and minor complex IV-containing supercomplex of unknown composition; an explanation is provided in the text (Section 2.3). The amount of dimers III_2_ in SC_1_ and SC_2_ was clarified by comparing the masses of the supercomplex SC_2_ of the three studied species and potato tuber supercomplex I_2_III_2_, all similarly migrating in BN-gel (Figure S2). The structure of the potato assembly was revealed earlier using single particle electron microscopy [8].

A comparison of the OXPHOS structures solubilized from mitochondria of these species revealed a significant similarity in their OXPHOS organization (Figure 1). Recently, we thoroughly studied the complement of the OXPHOS components in pea mitochondria using BN-related techniques in combination with mass spectrometry and immunochemical analyses [19]. Taking into account the observed similarity in profiles of the BN-resolved OXPHOS components and conserved arrangement of supercomplexes [11], it was concluded that the studied species have the following common OXPHOS structures: megacomplex (II_x_III_y_IV_z_)_n_, complex I-containing supercomplexes SC_2,4–6_, supercomplexes III_2_IVa_1–2_, dimers IV_2_ and Vb_2_, as well as monomeric forms IVa/b and Va/b. Further detailed proteomic and structural investigations will clarify exact stoichiometry and species-related structural features of the revealed supercomplexes. In the current study, species-specific differences in detergent-stability and activity of the OXPHOS components were observed using 1D BN-PAGE followed by in-gel enzyme activity staining and densitometry analysis (Figure 1). 

### 2.1. Relative Abundance and Activity of Superassembled and Free Complex I in Mitochondria of Pea, Winter Wheat and Maize

The main activity of complex I in all three species was detected in supercomplexes and only minor activity was observed in monocomplex I, indicating that the major part of the enzyme population exists in assemblies in vivo (Figure 1I). As a result of digitonin treatment, six complex I-containing supercomplexes (SC_1–6_) were isolated from pea mitochondria; five (SC_1,2,4–6_) and four supercomplexes (SC_2,4–6_) were solubilized from the winter wheat and maize organelles, respectively. Thus, under the conditions applied, SC_1_ was found in pea and in trace amounts in winter wheat, but was absent in maize; SC_3_ was almost undetectable in cereals at the protein loading used. The mitochondria of all studied species contained common supercomplexes SC_2_ and SC_4–6_ with close apparent masses (Table S1). Complex I in all species had an apparent molecular weight of 1130 kDa. The NADH dehydrogenase activity of alternative NAD(P)H dehydrogenases, which was more pronounced in maize, was also detected in the lower part of the gel (Figure 1I). 

**Figure 2:**
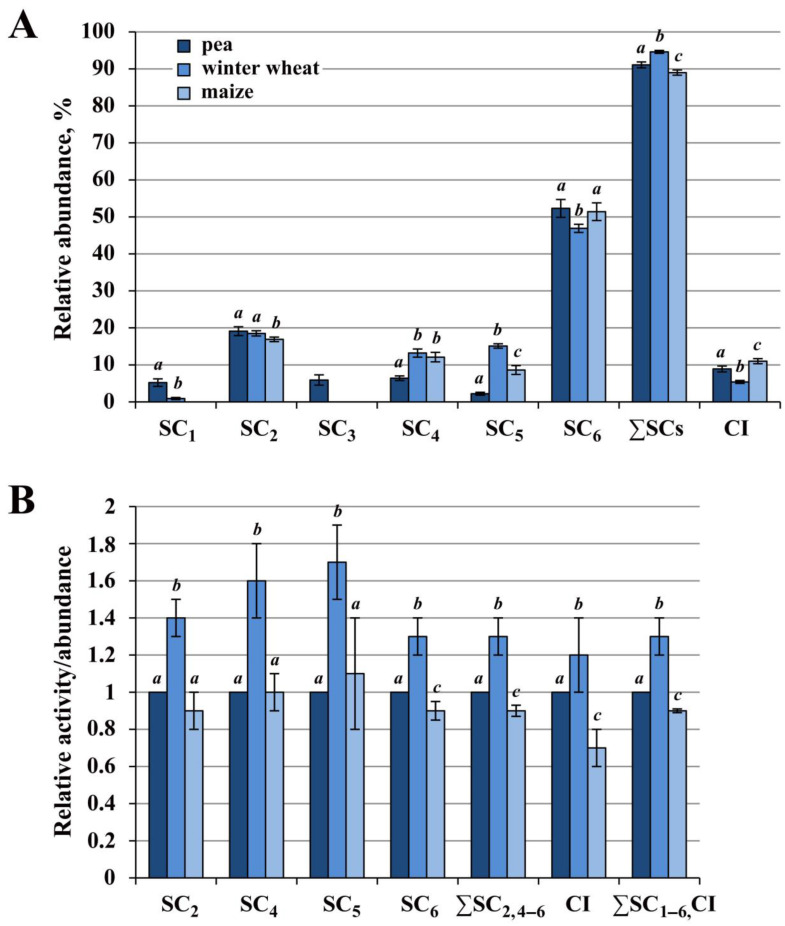
Relative abundance of supercomplexes SC_1–6_ and monocomplex I (**A**) and intrinsic activity of complex I (**B**) in mitochondria of pea, winter wheat, and maize. SC_1–6_—the putative composition of each supercomplex is given in the caption to Figure 1 and in Table S2. (**A**) The relative abundance of each supercomplex, free complex I (CI), and sum of all supercomplexes (∑SCs) was calculated based on densitometric analysis of Coomassie-stained gel as a percentage of the total intensity of all electrophoretic bands containing complex I (Table S2). (**B**) Comparative activity values, calculated as the ratio of relative activity to relative abundance, are presented (Table S3). The relative abundance and activity of pea were conventionally taken as 100%, and the ratio amounted to 1. The species color designations are the same as in the legend in panel A. The mean values and standard deviations are given. Identical letters indicate the absence of significant differences between the species for every analyzed OXPHOS structure or sum of indicated ones (at *p* ≤ 0.001).

As follows from the available literature data [34] and analysis of reference 2D BN/SDS gels (see https://gelmap.de), protein contamination in the high molecular weight region (≥1000 kDa) of the first dimension (1D BN-PAGE) is significantly lower than in the low molecular weight one where monocomplexes IV and II are separated. This made it possible to approximately estimate the relative abundance of supercomplexes SC_1–6_ (I_1–2_III_2_IV_0–n_) and individual complex I (Table S2). Densitometric analysis showed close ratios of major supercomplexes SC_2_ (I_2_III_2_) and SC_6_ (I_1_III_2_) in mitochondria of all three species. The ratio of SC_2_/SC_6_ in maize was 1:3, and in pea and wheat, it approached this value (Figure 2A; Table S2). Pea mitochondria differed from cereal ones by the presence of high-molecular respirasomes SC_1_ and SC_3_ as well as by a significantly lower proportion of basic respirasomes SC_4/5_ (I_1_III_2_IVa/b). A significant difference was observed between the cereals themselves in the abundance of supercomplexes SC_5_ and SC_6_. Major SC_6_ in winter wheat was, on average, 4.5% lower, and respirasome SC_5_ was 1.8 times higher compared maize. The latter fact may indicate the high detergent resistance of the respirasome SC_5_ (I_1_III_2_IVb) in winter wheat.

The close ratios of major supercomplexes SC_2_ (I_2_III_2_) and SC_6_ (I_1_III_2_) suggest a similar architecture of mitochondrial OXPHOSs in the studied species in vivo. A significant amount of the core supercomplex I_1_III_2_ may be the result of digitonin-caused breakdown of larger supercomplexes. The trace amounts or absence of high-molecular-weight SC_1_ (I_2_III_2_IV_n_) and SC_3_ (I_1_III_2_IV_n_) in combination with the high abundance of smaller respirasomes SC_4/5_ (I_1_III_2_IVa/b) in solubilizates from cereal organelles may indicate greater lability of high-molecular-weight “heavy” supercomplexes SC_1_ and SC_3_ and/or a more highly organized OXPHOS in pea. 

Interestingly, the frost-resistant winter wheat was characterized by the lowest amount of core supercomplex I_1_III_2_ and the highest abundance of respirasome I_1_III_2_IVb. In addition, this cereal also had the lowest amount of free complex I, which was on average 1.6 and 2 times lower than in the mitochondria of pea and maize, respectively. These data suggest a stronger interaction of complex IV with the core supercomplex I_1_III_2_ and more superassembled complex I in winter wheat compared to the enzyme of the other two species. 

#### 2.1.1. Winter Wheat Complex I Had the Highest Activity

A comparison of the absolute values of the NADH:Nitro Blue Tetrazolium (NBT) in-gel activity showed that the total activity of complex I (∑SC_1–6_, CI) in winter wheat was 30% and 40% higher than that in pea and maize, respectively (Figure 2B; Table S3). In particular, in winter wheat, the activity of bound complex I (∑SC_2,4–6_) was on average 30% and 40% and the activity of free enzyme (CI) was 20% and 50% higher than those in pea and maize. Moreover, the NADH dehydrogenase activity of respirasomes SC_4/5_ from winter cereal was significantly higher (on average by 60–70%) than that of pea and maize. The obtained data agree well with previous reports concerning the respiratory activity of pea, winter wheat, and maize mitochondria oxidizing malate, complex I substrate [39]. The authors showed that winter wheat organelles in comparison with those from pea and maize were characterized by significantly higher rates of phosphorylating (V3) and non-phosphorylating (V4) respiration, namely during oxidation of malate. The observed relationship between the activity of complex I and the low-temperature tolerance of the studied species may indicate an important role of this enzyme in the mechanisms of cold adaptation. 

The revealed consistency of our data on NADH:NBT in-gel activity of complex I with the literature data on complex I-maintained respiratory activity in organello suggests that the in-gel activity assay could be used not only for the enzyme detection within gels, but also for a comparative preliminary quantitative assessment of the enzyme activity in different species. It should be noted that at present the mechanism of the NADH:NBT in-gel reaction is only hypothetical and not fully understood. At the same time, tetrazolium salts have been used to quantify the respiratory activity of microorganisms [40] and mitochondria [41]. It has been shown that they could be reduced to formazan not only by flavin sites of NAD(P)H and succinate dehydrogenases, but also by ubiquinone and ROS [40]. The recent high-resolution cryo-EM structures of both individual complex I [42] and supercomplex I_1_III_2_ [15] have revealed bound ubiquinone located in Q-binding cavity. This is not surprising, because the Q-binding site in complex I is long enough to accommodate nearly an entire quinone molecule [43]. In this regard, reduction of ubiquinone by the chain of Fe-S clusters of free and superassembled complex I and subsequent oxidation by NBT in the in-gel activity assay cannot be excluded (especially given the electron transfer rates from NADH to N2 of about 100 μs [43]). This assumption is in good agreement with data on rotenone inhibition of NBT reduction in both in-gel activity assays of wheat complex I [44] and complex I substrate-fueled respiratory activity measurements of mitochondria isolated from rat liver [41]. Nevertheless, it should be noted that data concerning rotenone inhibition of NBT reduction are rather limited and generally data on sensitivity of tetrazolium dyes to electron transport chain inhibitors are scarce and contradictory. In this connection, this issue deserves further closer examination and consideration. Earlier, we also described the difference in NADH:NBT in-gel activity between free and superassembled forms of complex I for pea mitochondria [19]. Thus, the differences in complex I activity in different states and species observed by histochemical staining of BN-gels prompt further investigations of this technique, which in combination with densitometry analysis could be a useful tool for at least preliminary quantification of the enzyme activity.

### 2.2. Pea complex II Was More Digitonin-Resistant Compared to Winter Wheat and Maize Enzyme

Detection of succinate dehydrogenase activity in 1D BN-gel showed that while the pea enzyme was solubilized mainly as a free holoenzyme with an apparent mass of 165 kDa, almost all of the complex II activity of cereals was detected in the region of 88 and 91 kDa (Figure 1II; Table S1). A weak minor band of the activity with the close mass of 90 kDa was also detected in pea, which, as previously suggested [19], represented the holoenzyme breakdown product. This subcomplex is close in mass to the subcomplex IIa of *Arabidopsis* succinate dehydrogenase with an apparent mass of 94 kDa [45]. Subcomplex IIa is the largest dissociation product of complex II destabilized by low concentrations of dodecylmaltoside and is a matrix-exposed domain consisting of SDH1 (flavoprotein) and SDH2 (iron-sulfur protein) subunits. Since orthologs of these subunits have a high degree of amino acid sequence similarity in the dicotyledonous and monocotyledonous plant species studied [46], one can assume that the major activity bands in monocotyledonous winter wheat and maize also contain a matrix-exposed subcomplex including SDH1 and SDH2. Unlike winter wheat, a further barely visible band with a mass of 138 kDa, which could be a more preserved part of the enzyme, was detected in maize. In addition to free holoenzyme in pea and subcomplexes in cereals, a high-molecular-weight megacomplex (II_x_III_y_IV_z_)_n_ was found in all three species, which was the most abundant in pea solubilizates (Figure 1II). It was present in trace amounts in winter wheat and maize samples. Thus, the data obtained indicate higher stability of free and superassembled complex II in pea and its higher digitonin sensitivity in cereal organelles.

### 2.3. Most of the Complex IV Population in All Species Was Digitonin-Sensitive and Solubilized As Free Monomers and Dimers

Under the experimental conditions applied, the activity of complex IV in the studied species was detected in a number of the OXPHOS structures: megacomplex (II_x_III_y_IV_z_)_n_, respirasomes I_1–2_III_2_IV_1–n_, smaller supercomplexes III_2_IVa_2_ and III_2_IVa/b, and free dimeric IV_2_ and monomeric IVa and IVb forms (Figure 1IV). It should be noted that cytochrome *c* oxidase activity in the SC_2_ region in cereals, unlike pea, was detected in trace amounts. With similar apparent masses (Table S1) and close amounts of the SC_2_ band (Table S3) in all the species, this may indicate not only a higher enzyme activity in pea but also comigration of two different supercomplexes, a major I_2_III_2_ and unknown complex IV-containing minor supercomplex, in this region rather than one respirasome I_2_III_2_IV_n_. The latter minor supercomplex obviously is more abundant in pea. 

**Figure 3:**
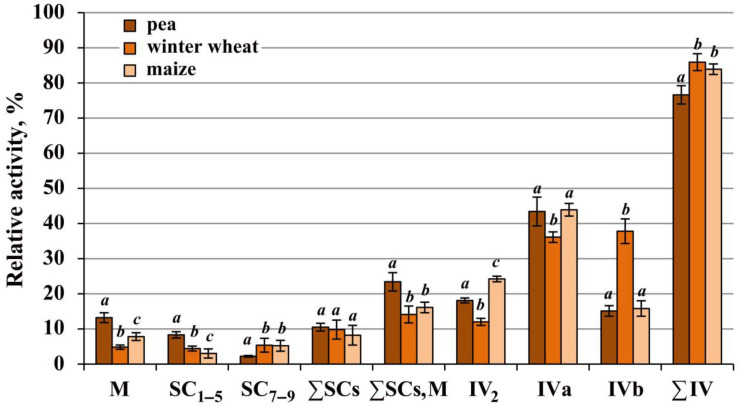
Relative activity of superassembled and free complex IV from mitochondria of pea, winter wheat, and maize shoots. M—megacomplex (II_x_III_y_IV_z_)_n_; SC_1–5_, SC_7–9_—to facilitate the perception of the material, the total activity of respirasomes SC_1–5_ and small supercomplexes SC_7–9_ is presented; the composition of supercomplexes SC_1–5_ is indicated in the caption of Figure 1; SC_7–9_: SC_7_—III_2_IVa_2_, SC_8—_III_2_IVa, SC_9_—III_2_IVb; ∑SCs—total activity of complex IV in supercomplexes SC_1–5_ and SC_7–9_; ∑SCs, M—total activity of the enzyme in supercomplexes SC_1–5,7–9_ and the megacomplex; ∑IV—the total activity of free dimers IV_2_ and monomers IVa and IVb. The mean values and standard deviations are given. Identical letters indicate the absence of significant differences between the species (at *p* ≤ 0.001). The numeric values are given in Table S4.

Since accurate quantification of complex IV forms on Coomassie-stained 1D BN-gels was difficult due to a significant number of other proteins, especially in IV_2_, IVa, and IVb regions, the relative abundance of the enzyme forms was estimated indirectly by their activity. The main activity of complex IV (on average, from 76.7% in pea and up to 84.0–85.7% in cereals) was observed in free dimeric IV_2_ and monomeric IVa and IVb forms (Figure 3), which suggested that the native enzyme assemblies are particularly detergent-sensitive. The results showed that the ratio of these forms varied in the studied species (Figure 1 and Figure 3, Table S4). Thus, in pea and maize, the main solubilized form of free cytochrome *c* oxidase was the form IVa. At the same time, the proportion of dimer IV_2_ was higher in maize, which may indicate its greater stability in the mitochondria of the heat-loving cereal. Winter wheat was characterized by a close ratio of IVa and IVb monomers, with the highest amount (among the species) of the basic form IVb and the lowest one of the dimer IV_2_, suggesting a greater digitonin sensitivity of the enzyme in this plant. The apparent masses of free forms of complex IV also differed somewhat between the species (Figure 1IV; Table S1). The winter wheat enzyme had the lowest apparent molecular weights and complex IV of maize occupied an intermediate position between pea and wheat under the experimental conditions used.

Among the species studied, the highest proportion of the total activity of bound (∑SCs, M) enzymes and simultaneously the lowest of that of free (∑IV) enzymes were observed in pea, which was caused by the higher abundance of cytochrome *c* oxidase in the megacomplex (Figure 3, Table S4). The total amount of the rest of the supercomplexes (∑SCs) did not differ between the species. At the same time, the proportion of respirasomes SC_1–5_ in pea was significantly higher than in cereals, while the latter were characterized by a higher abundance of small supercomplexes SC_7–9_ (III_2_IVa/b_1–2_). This may be due either to the greater detergent sensitivity of large respirasomes or to the peculiarities of the OXPHOS organization in cereals. A comparison of the total intrinsic activities of complex IV between the species demonstrated that the pea enzyme was, on average, 17.4% and 26.8% more active than the winter wheat and maize enzymes, respectively (Figure S3). 

### 2.4. Complex V of Cereals Was Highly Stable, Whereas the Pea Enzyme Was More Active

**Figure 4:**
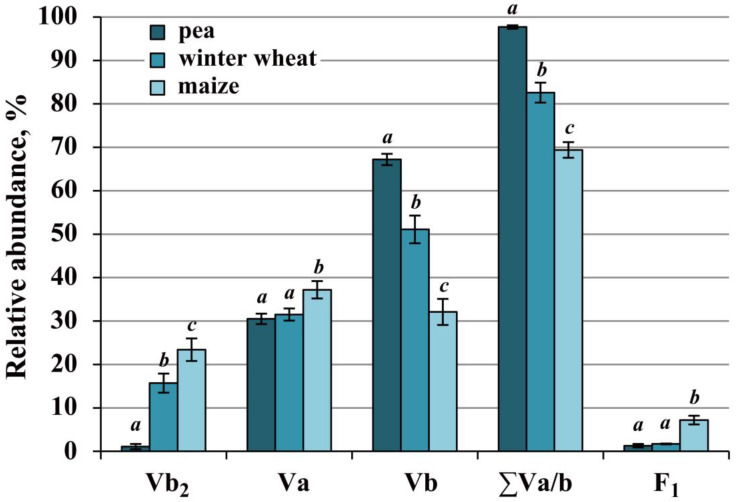
The relative abundance of complex V forms in solubilizates from pea, winter wheat, and maize mitochondria. Va, Vb, Vb_2_—monocomplexes Va/b and dimer Vb_2_; ∑Va/b—total relative abundance of monocomplexes Va and Vb; F_1_—component F_1_ of ATP synthase. The mean values and standard deviations are given. Identical letters indicate the absence of significant differences between the species (at *p* ≤ 0.001). The numeric values are given in Table S5.

The main part of the population of ATP synthase in pea, winter wheat, and maize was solubilized as monomers Va and Vb (Figure 1, Figure 4 and Figure S1; Table S5). In addition to monomers, the dimer Vb_2_ and trace amounts of supercomplex IV_1_Va_2_ and subcomplex F_1_ were also detected in all species. In pea, the breakdown product F_1_^*^ of the F_1_ subcomplex and minor amounts of dimer Va_2_ were further detected, and in maize, the oligomer V_n_ migrated to the 2760 kDa region (just below pea and wheat SC_1_) (Figure 1V and Figure S1). All forms of complex V in the studied species had similar calculated apparent molecular weights (Table S1).

Thus, the complements of ATP synthase forms in pea, winter wheat, and maize were significantly similar, but the ratio of these forms after solubilization differed between the species (Figure 1 and Figure 4, Table S5). The highest total monomer amount (∑Va/b) was observed in pea and the lowest was in maize. Winter wheat had an intermediate abundance. In pea and winter wheat, the main solubilized form was the basic form Vb, while in maize, the ratio of the two monomers was close. The low content of the monomer Vb in maize compared to pea and winter wheat was apparently related to the preservation of a significant part of the enzyme population as dimer Vb_2_. This indicates the high stability of the dimeric form of maize ATP synthase under the conditions applied. Winter wheat was also characterized by a significant, although lower than in maize, amount of dimer Vb_2_. Moreover, the minor amount of subcomplex F_1_ and the absence of its dissociation product F_1_^*^ also indicate the high stability of the ATP synthase monomer in this cereal (Figure 1V, Figure 4 and Figure S1). The trace amounts of pea dimeric ATP synthase, the high abundance of monomers, and the presence of breakdown products F_1_^*^ suggest a higher digitonin sensitivity of the pea enzyme. At the same time, despite the lability, the total intrinsic activity of pea complex V was, on average, 25.6% and 37.7% higher than in winter wheat and maize, respectively (Figure 1V and Figure S3).

## 3. Discussion

### 3.1. The OXPHOS Architecture in Pea, Winter Wheat, and Maize Shoot Mitochondria has Conserved Features

The study of the OXPHOS composition in the mitochondria from etiolated shoots of pea, winter wheat, and maize showed that the OXPHOS in all three species included common superstructures, such as megacomplex (II_x_III_y_IV_z_)_n_; complex I-containing supercomplexes SC_2,4–6_ (I_1–2_III_2_IV_0–n_), of which the two major ones, SC_2_ and SC_6_, had close abundance ratios (≈1:3); and also small supercomplexes III_2_IV_1–2_. In addition, free dimers of cytochrome *c* oxidase and ATP synthase, as well as their monomeric forms IVa/b and Va/b, were present. The data suggest a similar OXPHOS architecture in the species’ mitochondria in vivo. The close ratios SC_6_/SC_2_ in pea, winter wheat, and maize (2.5–3) has led to an attractive speculation concerning their different subcompartmentalized localization in the inner mitochondrial membrane. Similar ratios were revealed for cristae and inner boundary membrane-located complexes III and ATP synthases (CM/IBM complexes = 2.2–2.6) [47]. Such a consistency of values could suggest that the major SC_6_ (I_1_III_2_) may be the main solubilized supercomplex of the cristae membrane, while SC_2_ (I_2_III_2_) may be localized in the inner boundary membrane. 

The obtained results demonstrate that the main part of the complex I population, on average about 90 to 95%, in the mitochondria of the three species was superassembled into supercomplexes (Figure 1I and Figure 2A; Table S2). Previously, other researchers have found that free complex I in digitonin solubilizates of mammalian mitochondria accounted for about 14–16% [48], whereas 10 to 50% of the unbound enzyme was isolated from organelles of various plant species [32,35,44,49]. Variations in the amount of free solubilized enzyme may be related to the higher lability of plant supercomplexes, to the degree of intactness of mitochondria used (discussed in [19,30]), or/and to the particular features of OXPHOS organization in the organism studied. Based on the available literature data, it was suggested that under physiological conditions in vivo, almost all of complex I was part of the supercomplexes [50]. This may be explained by the dependence of the complex I assembly and stability on the respirasome formation [23,51]. 

In contrast to complex I, the other OXPHOS complexes studied, namely succinate dehydrogenase, cytochrome *c* oxidase, and ATP synthase, were solubilized mostly in the free forms, consistent with other studies [32,35,44,46], and apparently resulted from a more labile assembly of these enzymes in vivo. The population of free cytochrome *c* oxidase in all three species was represented by three forms: dimeric IV_2_ and two monomeric forms, IVa and IVb, previously described in other plants (dimeric form [30]; monomeric form [32,36]). ATP synthase was solubilized as the Va/b_2_ dimers and Va/b monomers. The high-molecular-weight form Va was newly detected and analyzed in the pea shoot mitochondria [19] and represented an enzyme associated with proteins that have not yet been identified. The data obtained in the present research suggest that this form is typical for different plant species, at least at seedling stage. In addition to the main conserved features, the OXPHOS of each plant under the study also had its own specific ones, apparently related to the species life strategies. 

### 3.2. Distinctive Features of the OXPHOS in Frost-Tolerant Winter Wheat

The winter wheat oxidative phosphorylation system was distinguished by a number of particular features, namely by (i) a mostly superassembled and highly active complex I; (ii) highly stable basic respirasomes I_1_III_2_IVa/b and ATP synthase; (iii) more detergent-sensitive complexes II and IV under the applied solubilization conditions. The high yield of respirasomes I_1_III_2_IVa/b in this cereal was correlated with a lower amount of free complex I and core supercomplex I_1_III_2_ (Figure 2A; Table S2). These findings may indicate a highly superassembled state of complex I and increased physical interactions of respiratory complexes in winter wheat respirasomes. The latter conclusion is consistent with the earlier suggestion that the intensity of enzyme interactions in supercomplexes varies in different plants [31]. Thus, it was previously found that in mitochondrial solubilizates from the appendix of thermogenic *Arum maculatum* [52] and suspension cell culture of salt-tolerant *Cakile maritima* [35], almost all the population of complex I was found assembled in I_1_III_2_ supercomplexes. This may reflect a stronger physical interaction between respiratory complexes I and III_2_ in these plants. Based on our findings and the above-mentioned literature data, it can be assumed that in plants whose life program implements the development of tolerance to external or internal environmental factors, the OXPHOS is characterized by a high structural stability of its basic components such as respiratory supercomplexes I_1_III_2_ and I_1_III_2_IV. The increased stability of these assemblies apparently ensures proper operation of the OXPHOS in a changing environment, with the resulting reliable ATP production required for stress tolerance (viz., for biosynthesis of reducing agents, osmolytes, and stress proteins).

It has previously been proposed that the dimeric and tetrameric complex IV modules mediate the binding of supercomplexes into respiratory strings [8,24,25] or association of supercomplexes with oligomeric ATP synthase in oxphosomes [19]. The lower stability of complex IV dimers in frost-resistant winter wheat may indicate a higher dynamism of this linker, which could be necessary for rapid OXPHOS rearrangements in response to changes in external or internal environmental conditions.

The high basic activity of winter wheat complex I (Figure 2B; [39]) appears to be associated with the main contribution of this complex to mitochondrial respiration under optimal conditions. Furthermore, the relationship between low temperature tolerance and the enzyme activity in the studied species suggests its involvement in cold adaptation and survival mechanisms. This is also indicated by a significant increase in complex I substrate-fueled respiratory activity (V3 and especially V4 rates) in winter wheat mitochondria after cold shock treatment [39]. In addition, it was shown that cold-shock-induced complex I activity in the winter cereal was accompanied by powerful AOX activation. Such coordinated work of these enzymes apparently contributes to the maintenance of (i) the transmembrane potential at the inner mitochondrial membrane (due to the cooperative work of the first coupling point and AOX); (ii) the optimal ROS amount (due to the reduction in O_2_^·–^ generation by complexes I and III); (iii) the energy-dissipating thermogenic function of the alternative terminal oxidase. Thus, it seems that in winter wheat, under optimal conditions, complex I is the main entry point for electrons into the respiratory chain and under unfavorable low-temperature conditions, this enzyme, in cooperation with AOX, forms an important protective mechanism, stabilizing and supporting the functional activity of the species’ mitochondria.

### 3.3. OXPHOS Features in the C4 Plant Maize

Maize, a C_4_-type plant, was characterized by a lower activity of complex I (Figure 1 and Figure 2; Table S3) and the highest stability of cytochrome *c* oxidase and ATP synthase dimers (Figure 1, Figure 3 and Figure 4; Tables S4 and S5). The reduced activity of complex I in this plant was apparently compensated for by a higher activity of alternative NAD(P)H dehydrogenases. This could be due to an elevated abundance of the latter enzymes caused by the increased need for oxidation of excess reducing equivalents formed during active photosynthesis.

The high stability of cytochrome *c* oxidase and ATP synthase dimers may be related to both the higher structural stability of maize dimeric forms and the less fluid phospholipid environment of these proteins. Given the high stability of the maize ATP synthase dimer and the detergent sensitivity of the monomer (Figure 4; Table S5), one can assume that the stability of the former depended primarily on the unsaturation degree of surrounding phospholipids. It is known that *cis*-double bonds of unsaturated fatty acid chains of phospholipids reduce the packing of adjacent lipids and, thus, increase the membrane fluidity, ensuring the functional activity and mobility of membrane proteins [53]. Analysis of the literature data concerning the degree of fatty acid unsaturation of mitochondrial membrane lipids in winter wheat, maize, and pea revealed a clear inverse relationship between the level of unsaturation and the number of dimers remaining after solubilization. Thus, maize had, on average, 72% unsaturated fatty acids [54] and 23.4% dimers (Table S5); winter wheat had 81% [55] and 15.7%; pea had 92% [56] and 1.1%, respectively. It is likely that the higher saturation and, consequently, the tighter lipid packing in maize mitochondrial membranes contribute to the preservation of ATP synthase dimers during solubilization. The high mitochondrial membrane saturation, the stability of IV_2_ and V_2_ dimers, and the high activity of alternative NAD(P)H dehydrogenases appear to be important features of the maize OXPHOS, which could reflect the heat-loving nature of this plant and support successful chloroplast–mitochondrial interactions, enabling highly efficient photosynthesis.

### 3.4. High-Energy Pea OXPHOS

The phosphorylating system of mitochondria from etiolated pea shoots had the following features: (i) large respirasomes, I_2_III_2_IV_n_ (SC_1_) and I_1_III_2_IV_n_ (SC_3_), and more abundant megacomplex (II_x_III_y_IV_z_)_n_, (ii) stable succinate dehydrogenase, (iii) labile and at the same time highly active ATP synthase, as well as (iv) rather stable and more active cytochrome *c* oxidase. The presence of large respirasomes and more abundant megacomplex (II_x_III_y_IV_z_)_n_ in pea mitochondrial solubilizates suggests a higher stability of these structures in pea organelles and/or a more ordered OXPHOS organization in comparison with that of cereals. The higher-ordered architecture could contribute to greater efficiency of the phosphorylating system. The validity of this assumption is supported by the data on mitochondrial respiratory activity, which revealed a higher degree of oxidation and phosphorylation coupling in pea shoot mitochondria compared to that in winter wheat and maize organelles under respiration on all substrates used (Table S6; [39,57]).

The higher stability of succinate dehydrogenase found in dicotyledonous pea (compared to monocotyledonous cereals) is consistent with the data of Huang et al. [46]. The authors showed that complex II of dicotyledonous *Arabidopsis* was more digitonin-resistant than that of monocotyledonous rice and had a slightly different subunit composition. The rice enzyme, like the winter wheat and maize one, was dissociated into similar subcomplexes in the presence of the detergent. It can be assumed that the high detergent resistance of complex II is a specific feature of the Dicotyledonae. As is known, complex II is an important component of both the respiratory chain and tricarboxylic acid cycle. The high stability of this enzyme and the high degree of oxidation and phosphorylation coupling during respiration on succinate (Table S6; [39]) indicate the importance of succinate dehydrogenase in energy production in the pea shoot mitochondria. Thus, larger respirasomes, a tight coupling state in oxidation of all substrates, the high stability of complex II, and the high activities of complexes IV and V suggest a higher-ordered OXPHOS architecture and increased energy metabolism in organelles of this species.

### 3.5. Putative Oxphosomic Organization in the Mitochondria of the Studied Species

**Figure 5:**
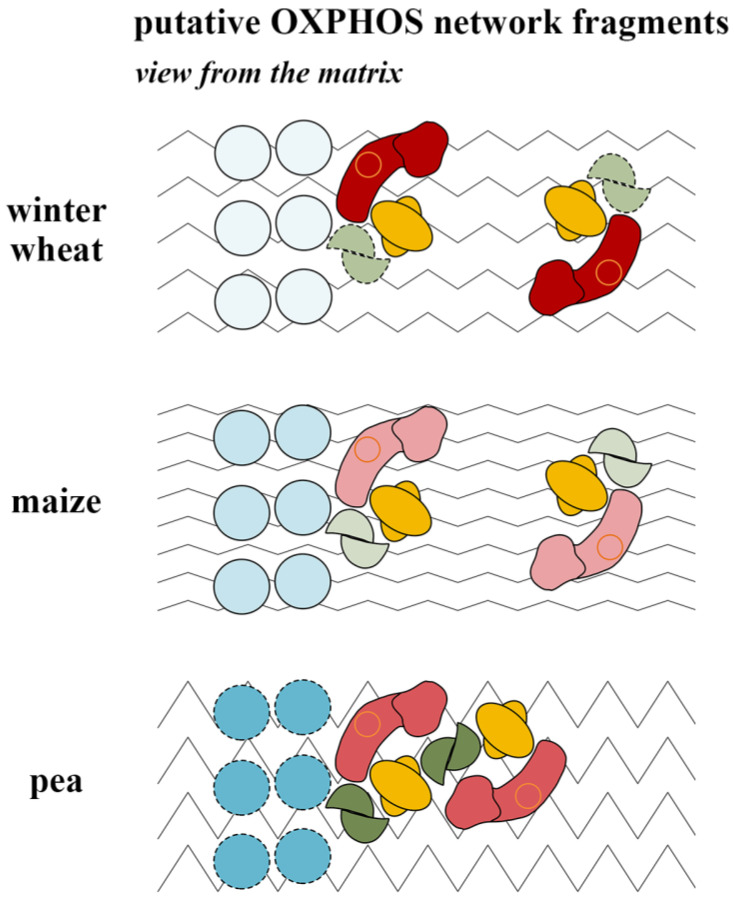
The putative organization of the OXPHOS network fragments in the mitochondria of etiolated shoots of winter wheat, maize, and pea from an oxphosomic point of view [19]. The assumed organization of the OXPHOS network fragments, consisting of the basic oxphosome I_1_III_2_IV_2_V_2_ (explanation of the stoichiometry in the text) and respirasome I_1_III_2_IV_2_, for mitochondria of each species of interest is presented. The matrix view is shown. Shades of red, yellow, green, and blue mean complexes I, III_2_, IV, and V, respectively. The depth of color indicates the activity level; the darker the color, the higher the activity. A dotted outline indicates unstable dimers of complexes IV (in winter wheat) and V (in pea). The degree of curvature of the zigzag lines schematically reflects the level of membrane lipid unsaturation; the steeper the zigzag, the higher the unsaturation (described in Section 3.3). The orientation of respirasomes relative to dimeric row ATP synthase is given taking into account the data of Nesterov et al. [59]. It is assumed that the pea OXPHOS has a higher-ordered arrangement, so that a greater number of supercomplexes are associated with higher-ordered structures. One of the possible variants of such higher-ordered OXPHOS structures is presented. The cereal OXPHOS seems more labile or/and more discrete and therefore the respirasomes I_1_III_2_IV_2_ are depicted separately from the basic oxphosomes I_1_III_2_IV_2_V_2_. The position of complex I relative to complex III_2_ is given based on the latest data on the structure of plant supercomplex I_1_III_2_ [15,16]. As a curious detail, it should be noted that apparently two populations of the supercomplex I_1_III_2_ coexist in the inner mitochondrial membrane. In the first one, the complex I has a left-sided position relative to dimer III_2_ (as in Figure 5, represented more widely in the literature), and in the second one, it has right-sided location (e.g., both arrangements, as well as their abundance, were shown by Gu et al. [9] (extended data Figure 4 in [9]).

Taking into account the overall similarity and the revealed species-specific features of the OXPHOS architecture and activity, functional and structural organization of the OXPHOS fragments in the studied species mitochondria was proposed from the point of view of the oxphosomic model (Figure 5) [19]. According to this model, the basic oxphosome has the composition I_1_III_2_IV_4_V_6_, which was assumed based on available data on the stoichiometry of the OXPHOS complexes, determined earlier for bovine heart mitochondria (I:II:III:IV:V ≈ 1:1.5:3:6:3) [48,58]. It has recently been demonstrated in reconstructions of inner mitochondrial membrane fragments from rat heart mitochondria that rows of respiratory supercomplexes could physically interact with rows of dimeric ATP synthases to form ordered oligomeric clusters [59]. It has been shown that the respirasomes tended to orient approximately along the row of ATP synthases and could dock with the latter through complexes IV or I. Still, despite the overall successful demonstration of ordered oligomeric clusters, it should be noted that the absence of IV_2_ dimers in the observed respirasomes and possibly even docking ATP synthase with complex I could be results of the partial disruption of the supramolecular structure of oligomeric clusters due to hypoosmotic shock during sample preparation.

Recent proteomic analysis of plant mitochondria isolated from the *Arabidopsis* cell culture (Dataset S3, worksheet #2 in [60]), obtained by LC-MS/MS, revealed that the stoichiometry of OXPHOS complexes (I:II:III:IV:V ≈ 1:1.5:3:1:3) is close to that of mammals. As can be seen from the available ratios, the main differences relate to the amount of cytochrome *c* oxidase, which, due to the high hydrophobicity of the enzyme subunits, is prone to be underestimated when using this method; in reality, it may be higher. Nevertheless, it is clear that the abundance of complex IV in plant mitochondria is significantly lower than in mammalian counterparts. Taking into account the correction for complex IV as well as cryo-electron tomography data [59], it is assumed that the basic plant oxphosome has a refined composition of I_1_III_2_IV_2_V_2_. The lower abundance of complex IV in plant mitochondria, which is supposed to be the main link between the respirasome and the ATP synthase dimer, is most likely due to the presence of alternative enzymes and may generally contribute to a more discrete (less superassembled) state of the plant OXPHOS compared to that in mammals.

## 4. Materials and Methods

### 4.1. Plant Material

The study was performed on etiolated shoots of 6-day-old pea seedlings (*Pisum sativum* L., cv. “Aksaiskiy Usatyi 55”, Don Zonal Research and Development Institute of Agriculture, Rassvet settlement, Rostov province, Russia) grown on wet pleated filter paper at 20 °C; 4-day-old etiolated shoots of winter wheat seedlings (*Triticum aestivum* L., cv. “Irkutskaya”, Siberian Institute of Plant Physiology and Biochemistry, Irkutsk, Russia) and maize (*Zea mays* L., cv. “Katerina”, LLC “Elevator”, Terek, Russia) were grown at 22 °C and 26 °C, respectively.

### 4.2. Isolation of Mitochondria

Mitochondria were isolated by differential centrifugation followed by purification at a stepwise gradient of Percoll density (23% and 40% (*v*/*v*)) as previously described [19]. For each isolation, 160 g of shoots was used and, finally, 12–16 mg of pure mitochondrial protein was solubilized. Centrifugation was carried out on an Allegra 64R centrifuge (Beckman Coulter, Brea, CA, USA). The purification time of the mitochondrial suspension on the Percoll gradient for pea was 35 min; for winter wheat and maize, it was increased to 40 min due to the smaller size of organelles. The purity and intactness of mitochondrial fractions were monitored by electron microscopy, outer mitochondrial membrane integrity tests [61], and polarographic analyses of organelle functional activity [19]. All tests showed the high purity, intactness, and functional activity of the isolated mitochondrial fractions, which resulted in the best suprastructure preservation and high respirasome yields under digitonin solubilization.

### 4.3. Digitonin Solubilization of Mitochondria

Freshly isolated mitochondria were resuspended in digitonin solubilization buffer at the ratio of 1 mg protein per 5 mg of digitonin in 100 μL of buffer (30 mM HEPES, 150 mM potassium acetate, 10% (*v*/*v*) glycerol, and 2 mM phenylmethylsulfonyl fluoride) as described before [19]. The advantage of using digitonin is mild solubilization and stabilization of the OXPHOS supercomplexes, resulting in higher yields of preserved superstructures and reproducibility of their relative abundance over a wide concentration range (5–10 g per g protein) [32,62]. This was not observed with harsher detergents (triton X-100 and dodecylmaltoside). The protein concentration in a suspension of freshly isolated mitochondria before solubilization was determined by a Bradford assay using bovine serum albumin as a standard. The solubilized samples obtained were stored at −80 °C. Coomassie G-250 dye was added immediately before electrophoresis.

### 4.4. One-Dimensional BN-PAGE and in-Gel Activity Staining of OXPHOS Complexes

Further resolving and analysis of the solubilized native proteins and their associations were performed using 1D BN-PAGE in a 3.5–16% polyacrylamide gradient gel (detailed in [19]). For equal protein loading (80 μg protein per well), the protein concentration in the obtained solubilizates was additionally measured by a Bradford assay (Table S7). To this end, samples were diluted 50 times to reduce the digitonin concentration to 0.1%, since concentrations above 0.1% could interfere with accurate measurements. The evenness of protein loading was additionally monitored by total track intensities on Coomassie-stained gels using ImageJ2x software (Table S7). The total intensity was very close in all analyzed samples. The obtained gels were either stained with a Coomassie R-250 solution or used for in-gel activity assays for complexes I, II, IV, and V, as described previously [19]. Histochemical staining of complex III in BN-gels was not performed due to the difficulty of its detection in supercomplexes [63]. However, the presence of complex III in supercomplexes and as an individual complex was clearly shown in our previous study of the OXPHOS organization in pea shoot mitochondria [19]. The high similarity of migration profiles of the OXPHOS components from all three species used suggested similar compositions of their OXPHOS supercomplexes. The Coomassie-stained gels and the gels with developed color enzymatic reaction (activity assay) were scanned on an Epson Perfection V700 Photo scanner and were quantified using ImageJ2x software. In total, for the reliable data analysis, nine BN-gels with equal protein loading of samples from nine different BN-PAGE runs were used. Additionally, three BN-gels were previously used to accurately align the protein loading of samples.

### 4.5. Determination of Molecular Weights of OXPHOS Supercomplexes and Complexes

To calculate the apparent molecular weights of the OXPHOS components isolated from the mitochondria of interest, bovine heart OXPHOS supercomplexes and complexes, solubilized with digitonin from bovine heart tissue (protein to detergent ratio of 1:5), were used as standard proteins [64] (Figure S2). The set of standards included respirasome I_1_III_2_IV_1_ (1700 kDa), supercomplex I_1_III_2_ (1500 kDa), complex I (1000 kDa), ATP synthase (750 kDa), complex III (500 kDa), complex IV (200 kDa), complex II (130 kDa) [62]. The mass calculation was carried out in Image Lab Software (Bio-Rad, Hercules, CA, USA, version 5.2.1).

### 4.6. Statistical Analysis

The results of the densitometric analysis are presented as mean values and standard deviations for two well-reproducible independent experiments (two mitochondrial isolations, purifications, solubilizations) with three technical repetitions (three independent BN-PAGE runs) for each assay and with a total of six resultant analytical repeats for each species and assay. The significance of differences was determined using an analysis of variance with the Student–Newman–Keuls method. For statistical processing, SigmaPlot 12.5 software was used.

## 5. Conclusions

In general, this study’s results revealed a highly conservative organization of mitochondrial phosphorylating systems in etiolated shoots of pea, winter wheat, and maize. The species had similar OXPHOS components and close relative abundance ratios of major supercomplexes I_2_III_2_ (SC_2_) and I_1_III_2_ (SC_6_) approaching the value of 1:3. Obviously, the latter fact indicates the similarity in the arrangement of the superstructures in vivo and their different subcompartmented location (discussed in Section 3.1). At the same time, the obtained data also revealed the specific OXPHOS features apparently related to the life strategies of each species. Thus, the OXPHOS of frost-resistant winter wheat was characterized by the highly stable core respirasomes I_1_III_2_IVa/b and a highly active complex I that probably allows this cereal to adapt to and function effectively under low-temperature stress conditions. At the same time, the detergent sensitivity of complex IV may reflect the lability of this putative linker in higher-ordered structures and the possibility of their rapid rearrangements in response to environmental factors. As noted above, maize, the C_4_ heat-loving plant, has a much narrower range of temperature adaptation in contrast to winter wheat but intense photosynthesis. In this regard, it can be assumed that the native architecture of the maize OXPHOS is not intended for active rearrangements in a natural environment, which correlates with the high detergent stability of complex IV and V dimers and the high saturation (i.e., lower fluidity) of mitochondrial membranes in this cereal. In addition, the compensatory higher capacity of alternative NAD(P)H dehydrogenases is obviously necessary for successful chloroplast–mitochondrial interactions during intensive photosynthesis, as it enables substantial re-oxidation of the resulting excess of NAD(P)H. The OXPHOS of nitrogen-fixing pea presumably has a higher-order organization in vivo, highly active ATP synthase and cytochrome *c* oxidase, stable succinate dehydrogenase, and more coupling state of oxidation and phosphorylation. All these peculiarities indicate a highly active and efficient energy metabolism in pea shoot mitochondria. The revealed features of OXPHOS organization and activity could be provided by an appropriate species-specific conformation of OXPHOS enzymes and/or their assemblies in vivo, which needs to be clarified by further biochemical and structural investigations.

## Data Availability

Not applicable.

## Supplementary Materials

The supporting information can be downloaded at: https://www.mdpi.com/article/10.3390/ijms242015229/s1.
