# Supplementary material for: OXPHOS Organization and Activity in Mitochondria of Plants with Different Life Strategies
Source: Int J Mol Sci. 2023 Oct 16;24(20):15229. doi: 10.3390/ijms242015229 (PMC10607765; doi:10.3390/ijms242015229)
Supplement: Supplementary file 1 [file ijms-24-15229-s001.zip › ijms-2617637-supplementary.pdf]

Table S1. Apparent masses of the OXPHOS complexes and supercomplexes from pea, winter wheat, and maize shoot mitochondria.

OXPHOS enzymes	Designations	Proposed composition	Calculated apparent mass, kDa		
			Pea	Winter wheat	Maize
Complex I	SC_1	$I_2III_2IV_n$	*2830*	*2790*	–
	SC_2^*	$I_2III_2+SC^{IV}$	*2350*	*2356*	*2350*
	SC_3	$I_1III_2IV_2$	*2060*	–	–
	SC_4	I_1III_2IVa	*1830*	*1810*	*1810*
	SC_5	I_1III_2IVb	*1750*	*1744*	*1744*
	SC_6	$I_1III_2+IV_1Va_2$	1630	1600	1600
	I		1130	1130	1130
Complex II	M	$(II_xIII_yIV_z)_n$	*3600*	*3590*	*3590*
	II_{major}		165	88	91
	II_{minor}		90	–	138
Complex III_2			500	485	490
Complex IV	M	$(II_xIII_yIV_z)_n$	*3600*	*3590*	*3590*
	SC_1	$I_2III_2IV_n$	*2830*	*2790*	–
	SC_2^*	$I_2III_2+SC^{IV}$	*2350*	*2356*	*2350*
	SC_3	$I_1III_2IV_2$	*2060*	–	–
	SC_4	I_1III_2IVa	*1830*	*1810*	*1810*
	SC_5	I_1III_2IVb	*1750*	*1744*	*1744*
	SC_6	$I_1III_2+IV_1Va_2$	1630	1600	1600
	SC_7	III_2IVa_2	1260	1205	1205
	SC_8	III_2IVa	900	884	884
	SC_9	III_2IVb	nd	790	790
	IV_2		400	340	360
	IVa		300	254	260
	IVb		200	183	190
Complex V	Vb_2		1330	1300	1315
	Va		780	770	785
	Vb		700	690	705
	F_1		290	300	310
Alternative NAD(P)H dehydrogenases	ND_{major}		111	116	114
			105	–	–

Designations: Complexes I, II, III_2, IV, and V—individual complexes I, II, III_2, IV, and V, as well as supercomplexes containing them and corresponding subcomplexes (where indicated); SC—supercomplex; M—megacomplex; $II_{major/minor}$—major and minor forms of complex II; ND_{major}—major forms of alternative NAD(P)H dehydrogenases. SC_2^*—instead of the previously assumed respirasome $I_2III_4IV_n$, two associations could comigrate in this band: I_2III_2 and minor complex IV-containing supercomplex of unknown composition (discussed in more detail in the caption of Figure 1 and in Subsection 2.3). The calculated masses of supercomplexes above 1700 kDa, i.e., above the highest molecular weight standard (Subsection 4.5), are given in italics because the absence of corresponding standards in the highest molecular weight region does not make it possible to accurately estimate their molecular weights [19]. The rounded mean values based on analysis of 15 BN-gels are presented.

Table S2. Relative abundance and NADH-dehydrogenase activity of supercomplexes $SC_{1–6}$ and monocomplex I in digitonin solubilizates of pea, winter wheat, and maize shoot mitochondria.

Estimated parameters	Designation	Putative composition	Pea	Winter wheat	Maize
Relative abundance, %	SC_1	$I_2III_2IV_n$	$5.2^{a} \pm 1.0$	$0.9^{b} \pm 0.3$	–
	SC_2	I_2III_2+SC^{IV}	$19.1^{a} \pm 1.2$	$18.5^{a} \pm 0.7$	$16.9^{b} \pm 0.6$
	SC_3	$I_1III_2IV_n$	5.9 ± 1.4	–	–
	SC_4	I_1III_2IVa	$6.4^{a} \pm 0.6$	$13.2^{b} \pm 1.1$	$12.1^{b} \pm 1.3$
	SC_5	I_1III_2IVb	$2.2^{a} \pm 0.4$	$15.1^{b} \pm 0.6$	$8.6^{c} \pm 1.2$
	SC_6	I_1III_2+IV_1Va_2	$52.3^{a} \pm 2.4$	$46.9^{b} \pm 1.1$	$51.4^{a} \pm 2.4$
	$\sum SC_{1–6}$		$91.1^{a} \pm 0.8$	$94.6^{b} \pm 0.4$	$89.0^{c} \pm 0.7$
	CI		$8.9^{a} \pm 0.8$	$5.4^{b} \pm 0.4$	$11.0^{c} \pm 0.7$
	SC_6/SC_2		2.7	2.5	3
Relative activity, %	SC_1	$I_2III_2IV_n$	$6.1^{a} \pm 1.0$	$1.1^{b} \pm 0.2$	–
	SC_2	I_2III_2+SC^{IV}	$21.0^{a} \pm 1.6$	$21.4^{a} \pm 1.2$	$19.4^{b} \pm 0.9$
	SC_3	$I_1III_2IV_n$	4.5 ± 0.9	–	–
	SC_4	I_1III_2IVa	$5.3^{a} \pm 0.4$	$12.8^{b} \pm 0.4$	$11.3^{c} \pm 1.3$
	SC_5	I_1III_2IVb	$1.6^{a} \pm 0.2$	$13.5^{b} \pm 0.2$	$7.3^{c} \pm 0.7$
	SC_6	I_1III_2+IV_1Va_2	$49.5^{a} \pm 2.4$	$44.3^{b} \pm 1.8$	$50.3^{a} \pm 2.1$
	$\sum SC_{1–6}$		$87.9^{a} \pm 2.0$	$93.2^{b} \pm 0.9$	$88.4^{a} \pm 0.7$
	CI		$12.1^{a} \pm 2.0$	$6.8^{b} \pm 0.9$	$11.6^{a} \pm 0.7$

$\sum SC_{1–6}$—sum of values for SC_{1-6}. Relative abundance or activity of each supercomplex and monocomplex I was calculated as a percentage of total intensity (total integrated density) of all complex I-containing bands. SC_6/SC_2—relative abundance ratio of supercomplexes SC_6 and SC_2. The mean values and standard deviations are given. The same letters indicate the absence of significant differences between the species for every analysed structure (or sum of them); different letters show statistically significant differences ($P \leq 0.001$).

Table S3. Activity of winter wheat and maize complex I in comparison with that of pea enzyme.

Estimated parameters	Designation	Pea	Winter wheat	Maize
Relative abundance, %	SC_2	100^a	$88.3^b \pm 9.2$	$86.7^b \pm 7.8$
	SC_4	100^a	$188.0^b \pm 17.4$	$185.9^b \pm 17.9$
	SC_5	100^a	$637.5^b \pm 97.8$	$398.3^c \pm 113.0$
	SC_6	100^a	$82.1^b \pm 5.3$	$96.9^c \pm 8.4$
	$\sum SC_{2, 4–6}$	100^a	$106.9^b \pm 6.4$	$109.3^b \pm 6.5$
	CI	100^a	$56.7^b \pm 9.8$	$122.9^c \pm 17.3$
	$\sum SC_{1–6}$+CI	100^a	$91.4^b \pm 4.8$	$98.2^a \pm 3.6$
Relative activity, %	SC_2	100^a	$123.3^b \pm 15.3$	$81.3^c \pm 10.7$
	SC_4	100^a	$292.4^b \pm 33.9$	$188.3^c \pm 24.9$
	SC_5	100^a	$1042.8^b \pm 149.1$	$407.1^c \pm 38.6$
	SC_6	100^a	$107.5^a \pm 7.1$	$89.0^b \pm 8.0$
	$\sum SC_{2, 4–6}$	100^a	$143.0^b \pm 9.8$	$99.9^a \pm 5.9$
	CI	100^a	$68.6^b \pm 7.7$	$86.0^c \pm 15.3$
	$\sum SC_{1–6}$+CI	100^a	$120.0^b \pm 6.0$	$87.3^c \pm 3.4$
Activity/abundance	SC_2	1^a	$1.4^b \pm 0.1$	$0.9^a \pm 0.1$
	SC_4	1^a	$1.6^b \pm 0.2$	$1.0^a \pm 0.1$
	SC_5	1^a	$1.7^b \pm 0.2$	$1.1^a \pm 0.3$
	SC_6	1^a	$1.3^b \pm 0.1$	$0.9^c \pm 0.05$
	$\sum SC_{2, 4–6}$	1^a	$1.3^b \pm 0.1$	$0.9^c \pm 0.03$
	CI	1^a	$1.2^b \pm 0.2$	$0.7^c \pm 0.1$
	$\sum SC_{1–6}$+CI	1^a	$1.3^b \pm 0.1$	$0.9^c \pm 0.01$

For comparison, abundance, activity, and the activity/abundance ratio of pea enzyme were taken as 100% and unit, respectively. The designations are the same as in Table S2. $\sum SC_{2, 4–6}$, $\sum SC_{1–6}$+CI—comparison of total abundance, activity or activity/abundance values for $SC_{2, 4–6}$ or supercomplexes $SC_{1–6}$ and monocomplex I, respectively. The mean values and standard deviations are given. The same letters indicate the absence of significant differences between the species for every evaluated OXPHOS component (or their sum). Different letters show statistically significant differences ($P \leq 0.001$).

Table S4. Relative activity of free and superassembled complex IV solubilized from pea, winter wheat, and maize mitochondria.

CIV	Pea	Winter wheat	Maize
M	12.8[a] ± 1.2	4.5[b] ± 0.7	7.9[c] ± 0.8
$SC_{1–5}$	8.3[a] ± 0.9	4.4[b] ± 0.7	3.0[c] ± 1.3
$SC_{7–9}$	2.2[a] ± 0.2	5.3[b] ± 2.0	5.2[b] ± 1.5
∑SCs	10.5[a] ± 1.1	9.8[a] ± 2.7	8.2[a] ± 2.8
∑SCs, M	23.3[a] ± 2.1	14.3[b] ± 3.3	16.0[b] ± 2.2
IV_2	17.4[a] ± 0.3	11.8[b] ± 0.7	24.3[c] ± 0.5
IVa	45.4[a] ± 3.6	34.8[b] ± 0.6	43.8[a] ± 1.1
IVb	13.9[a] ± 1.9	39.1[b] ± 4.2	15.9[a] ± 2.7
∑IV	76.7[a] ± 2.1	85.7[b] ± 3.3	84.0[b] ± 2.2

Designations are the same as in Table S2. CIV—complex IV activity in megacomplex, supercomplexes $SC_{1–5}$, $SC_{7–9}$ and dimeric IV_2 and monomeric IVa/b forms. $SC_{1–5}$, $SC_{7–9}$, ∑SCs and ∑SCs, M—total cytochrome *c* oxidase activity in $SC_{1–5}$, $SC_{7–9}$, all supercomplexes or supercomplexes and megacomplex, respectively. ∑IV—total activity of dimer IV_2 and monomers IVa/b. The mean values and standard deviations are given. The same letters indicate the absence of significant differences between the species; different letters show statistically significant differences ($P \leq 0.001$).

Table S5. Relative abundance of complex V forms solubilized from pea, winter wheat, and maize mitochondria.

CV forms	Pea	Winter wheat	Maize
Vb_2	$1.1^a \pm 0.6$	$15.7^b \pm 2.2$	$23.4^c \pm 2.6$
Va	$30.5^a \pm 1.2$	$31.5^a \pm 1.4$	$37.2^b \pm 2.0$
Vb	$67.2^a \pm 1.3$	$51.1^b \pm 3.2$	$32.1^c \pm 3.0$
∑Va/b	$97.7^a \pm 0.4$	$82.6^b \pm 2.3$	$69.4^c \pm 1.8$
F_1	$1.3^a \pm 0.4$	$1.7^a \pm 0.1$	$7.2^b \pm 1.0$

Complex V forms visible on Coomassie-stained BN-gels were analyzed. Vb_2—dimer of form Vb; Va and Vb—monocomplexes Va/b; ∑Va/b—sum of values for forms Va/b; F_1—subcomplex F_1 of ATP synthase. Component F_1 from pea mitochondria dissociated further into components, F_1^*, which were not taken into account due to the difficulty of detection on Coomassie-stained gels. The mean values and standard deviations are given. The same letters indicate the absence of significant differences between the species; different letters show statistically significant differences ($P \leq 0.001$).

Table S6. Comparison of coupling states of mitochondria from etiolated pea, winter wheat, and maize shoots based on the available literature data.

Oxidation substrate	Plant species	RC	References
Malate	Pea	$3.69^{a} \pm 0.13$	Kolesnichenko et al., 2003
	Winter wheat	$3.37^{b} \pm 0.11$	
	Maize	$3.36^{b} \pm 0.32$	
Succinate	Pea	$2.15^{a} \pm 0.01$	Grabelnych et al., 2004
	Winter wheat	$1.47^{b} \pm 0.03$	
	Maize	$1.90^{c} \pm 0.23$	
NADH	Pea	$1.41^{a} \pm 0.07$	Grabelnych et al., 2004
	Winter wheat	$1.16^{b} \pm 0.04$	
	Maize	$1.21^{b} \pm 0.03$	

Oxidation substrates: 10 mM malate in the presence of 10 mM glutamate, 8 mM succinate in the presence of 5mM glutamate, and 1mM NADH with the addition 0.06 mM $CaCl_2$. The mean values of respiratory controls (RC) and standard deviations are given. The same letters indicate the absence of significant differences between the species; different letters show statistically significant differences ($P < 0.050$).

Table S7. Protein quantification and control of equal protein loading.

Species		Bradford assay		Track intensity (Image J), %
		**Protein concentration, μg/μl**	**Protein loading, μg/well**	
Pea	1	13.9[a] ± 1.3	77.8[a] ± 7.2	100[a]
	2	13.8[a] ± 1.7	80.3[a] ± 9.8	99.9[a] ± 2.3
Winter wheat	1	8.1[b] ± 1.6	77.4[a] ±15.9	100.4[a] ± 3.2
	2	8.3[b] ± 2.6	79.1[a] ± 25.1	99.2[a] ± 5.0
Maize	1	8.3[b] ± 2.4	82.8[a] ± 23.4	99.1[a] ± 4.0
	2	7.1[b] ± 1.4	77.9[a] ± 15.2	98.1[a] ± 0.8

Protein quantification via a Bradford assay and data on protein loading as well as track intensities are given. Equal loading control and final fine equalization of the samples were carried out by means of total track intensity measurements using Image J software. Two biological replicates for every species with total 8-10 technical (4-5 for every replicate) and 24-30 analytical repetitions (12-15 for every one) were performed for protein quantification. Two biological, well-reproducible replicates were used to place two analytical repetitions for each biological replicate on the same gel (6 tracks x 2 repetitions, the first repetition was intended for abundance and the second one for activity) and to properly analyze the relative abundance and activity of the assembled and free OXPHOS enzymes. Overall, three technical repetitions for each biological replicate and six analytical repetitions for each species (for abundance or activity estimation) were produced. For track intensity comparisons, one pea replicate was taken as 100%. The mean values and standard deviations are shown. The same letters indicate the absence of significant differences between the replicates and species; different letters show statistically significant differences at the $P \leq 0.001$ level.

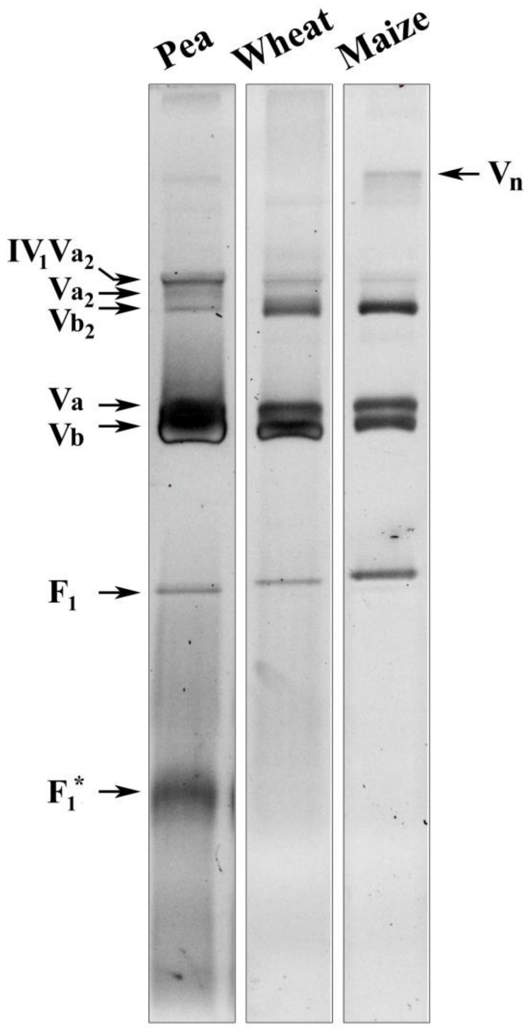

Figure S1. Digitonin-solubilized and BN-separated forms of complex V from pea, winter wheat, and maize shoot mitochondria. Results of in-gel ATPase activity staining are presented. Designations are the same as in Figure 1. The scanning was carried out using a Gel Doc Imaging system. In this scan mode, the ATPase activity of supercomplex IV_1Va_2 in the band SC_6 was visible not only in pea, but also in minor amounts in winter wheat and maize. In maize, additional ATPase activity was detected in the 2760 kDa region, close in mass to SC_1, which apparently could be an oligomeric form of ATP synthase (Vn).

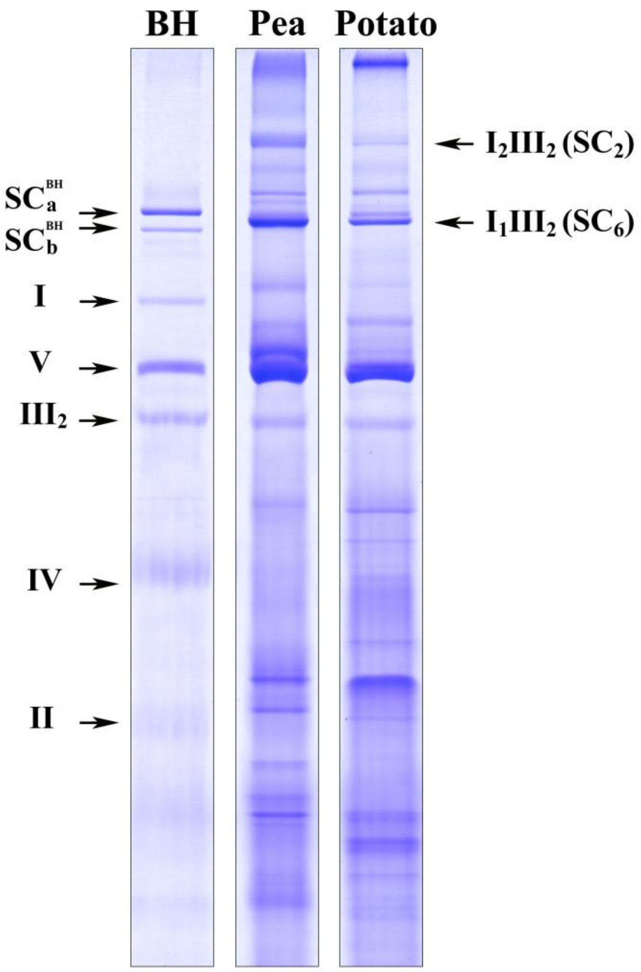

Figure S2. The complex I-containing supercomplexes of potato tubers and pea mitochondria have similar apparent molecular masses. BH—bovine heart OXPHOS supercomplexes and complexes used as standard proteins; SCa^{BH}, SCb^{BH}—supercomplexes $I_1III_2IV_1$ and I_1III_2 from bovine heart mitochondria, respectively; I, V, III_2, IV and II—corresponding OXPHOS complexes.

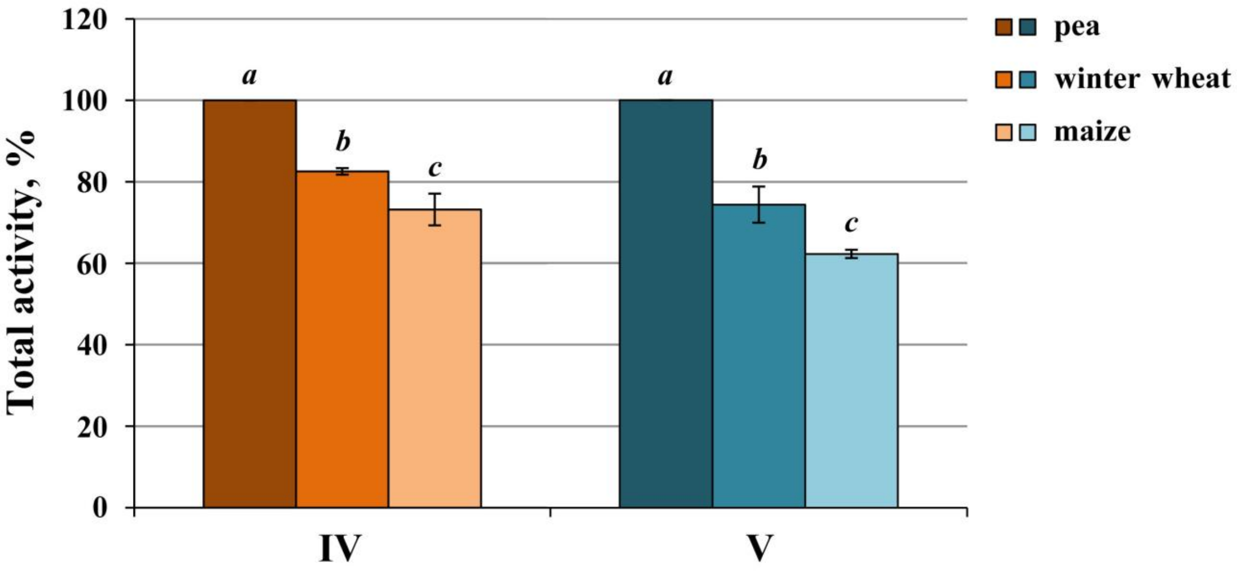

Figure S3. Total activity of mitochondrial cytochrome *c* oxidase and ATP synthase of winter wheat and maize in comparison with that of pea. IV—total activity of cytochrome *c* oxidase, which was calculated as sum of the integrated densities (IntDen) of complex IV activity bands (complex IV-containing megacomplex and supercomplexes, free IV_2, and forms IVa/b). V—total activity of ATP synthase, which was sum of integrated densities (IntDen) of Va/b and Vb_2 activity bands. The enzyme activity of pea was taken as 100%. For correct comparison, the complex V activity of winter wheat and maize was calculated for 100% abundance (of the complex V forms), normalized to pea. This was not done for the complex IV activity estimation due to difficulty of the enzyme's accurate quantification on 1D BN-gel (Subsection 2.3). The total complex IV activity for winter wheat was 82.6% ± 0.8%; for maize it was 73.2% ± 3.9% (relative to that of pea). Total complex V activity for winter wheat was 74.4% ± 4.4%; for maize it was 62.3% ± 1.0%. The mean values and standard deviations are given. Different letters indicate significant differences at the $P \leq 0.001$ level.
